# The transcriptomic profile of ovarian cancer grading

**DOI:** 10.1002/cam4.343

**Published:** 2014-10-15

**Authors:** Cindy Q Yao, Francis Nguyen, Syed Haider, Maud H W Starmans, Philippe Lambin, Paul C Boutros

**Affiliations:** 1 Informatics and Biocomputing Program, Ontario Institute for Cancer ResearchToronto, Ontario, Canada; 2 Department of Medical Biophysics, University of TorontoToronto, Ontario, Canada; 3 Computer Laboratory, University of CambridgeCambridge, United Kingdom; 4 Department of Radiation Oncology (Maastro), GROW-School for Oncology and Developmental Biology, Maastricht University Medical CenterMaastricht, The Netherlands; 5 Department of Pharmacology and Toxicology, University of TorontoToronto, Ontario, Canada

**Keywords:** Grading, linear modeling, microarray, ovarian carcinoma, serous subtype, survival analysis

## Abstract

Ovarian carcinoma is the leading cause of gynecological malignancy, with the serous subtype being the most commonly presented subtype. Recent studies have demonstrated that grade does not yield significant prognostic information, independent of TNM staging. As such, several different grading systems have been proposed to reveal morphological characteristics of these tumors, however each yield different results. To help address this issue, we performed a rigorous computational analysis to better understand the molecular differences that fundamentally explain the different grades and grading systems. mRNA abundance levels were analyzed across 334 total patients and their association with each grade and grading system were assessed. Few molecular differences were observed between grade 2 and 3 tumors when using the International Federation of Gynecology and Obstetrics (FIGO) grading system, suggesting their molecular similarity. In contrast, grading by the Silverberg system reveals that grades 1–3 are molecularly equidistant from one another across a spectrum. Additionally, we have identified a few candidate genes with good prognostic information that could potentially be used for classifying cases with similar morphological appearances.

## Introduction

Ovarian cancer is one of the most lethal gynecological cancers and is the fifth most common cause of cancer death in North America 1. Many subtypes of epithelial ovarian carcinoma exist including the serous, clear cell, endometrioid, and mucinous subtypes 2. There are substantial differences in genetic risk factors and somatic mutation profiles between each of these subtypes. The majority of ovarian carcinomas that are presented at the clinic correspond to cancer of the serous subtype 3.

Accurate diagnosis and prognosis are critical for disease management and therapeutics. To aid this, histopathological grade is intended to provide additional information to a nominal diagnostic category; information which should have prognostic or therapeutic implications. However, if designation of a tumor to a specific diagnostic category conveys sufficient information, grading is not necessary 4,5. Histologic reporting of ovarian carcinomas has traditionally required assessment of both cell type and grade. A number of grading systems exist, including the Silverberg 6, the International Federation of Gynecology and Obstetrics (FIGO) 7, the World Health Organization (WHO) 8, and the Gynecologic Oncology Group (GOG) systems 9. Each grading systems employs a different scheme, but most are ternary systems stratifying ovarian serous carcinomas into well, moderately, and poorly differentiated categories. These ternary grading systems imply a progressive deterioration in differentiation, and the Silverberg, FIGO, and WHO systems are “universal” in the sense that they can be applied regardless of cell type 6. The GOG system, by contrast, is cell-type–specific, requiring initial assessment of cell type with subsequent application of a histotype–specific grading system 9.

A two-tier system has been recently proposed, and is suggested to be superior to the above three-tier systems 10. Tumors classified into low- or high-grade ovarian carcinoma have distinct histological, molecular, and clinical profiles. Molecularly, low-grade serous carcinomas generally have low levels of chromosomal instability and carry frequent mutations in *KRAS*, *BRAF*, and *ERBB2*, while high-grade serous carcinomas tend to have high levels of chromosomal instability and frequently show mutations in *TP53* 10. Histologically, low-grade ovarian serous carcinoma generally has a micropapillary-rich growth pattern, while high-grade ovarian serous carcinoma adapts a large papillae and glandular pattern with infrequent micropapillary growth. Tumors classified by this binary grading system demonstrate diverse survival profiles, with median survival of 4.2 years in patients with low-grade tumors and 1.7 years in those with high-grade tumors 10,11.

Classification of some grade 2 tumors (characterized as having larger nuclei and nucleoli, coarser chromatin, and more mitotic activity) has been challenging 10,11. One of the significant aspects of accurate pathological grading is its association with treatment options. Since low- and high-grade tumors exhibit differences in proliferation rate, it is possible that they respond to chemotherapy differently 10; hence the accurate pathological grading of a tumor is exceptionally important. Previous clinical studies demonstrated that low-grade serous carcinoma were not as responsive to traditional chemotherapeutic agent, such as taxane and platinum, in comparison to high-grade carcinomas 12.

Although such grading lacks prognostic significance and clinical reproducibility, it remains possible that tumor grade can accurately capture some underlying molecular characteristics of the tumor that are not reflected through other measures 11. In fact, previous work using principal component analysis (PCA) on mRNA abundance profiles to dichotomize tumors into low- and high-grade groups 13. This strongly suggests the presence of clear molecular differences between tumors of different grades. To test this hypothesis, we surveyed the serous ovarian cancer transcriptome and identified genes associated with well, moderately, and poorly differentiated tumors as established by the FIGO and Silverberg systems. We assessed the association of these genes with patient survival and considered their involvement with known biomolecular pathways.

## Materials and Methods

### Patient cohort

Raw microarray data and patient-level annotation from multiple datasets were used 14–17. Raw data were assessed for distributional homogeneity. Redundant samples were identified by comparing raw array data (CEL files) across datasets and were excluded from the study. In addition, the large The Cancer Genome Atlas (TCGA) dataset 18 was not included in this study as it did not annotate which grading system was used, and the project spanned several years of reporting, hence it was likely that both grading systems were used at some centers. The remaining raw data were then loaded into R statistical environment (v2.15.3) using the affy package (v1.36.1). Probes were remapped to Entrez Gene IDs using the following packages: hgu95av2hsentrezgcdf v16.0.0, hgu133plus2hsentrezgcdf v16.0.0, hgu133ahsentrezgcdf v16.0.0. Data were preprocessed using the RMA algorithm 19 and associated with published patient annotation, including grade, primary tumor site, stage, survival status, and survival time. Patients that underwent neoadjuvant treatment prior to surgery were excluded from this analysis. A total of four datasets were employed: for each, the number of patients included, number of genes evaluated, and other clinical covariates are provided in Table1. To increase statistical power, we combined datasets based on their grading systems (i.e., datasets using the FIGO grading system were pooled, as were datasets using the Silverberg grading system). We first applied a Y-chromosome-based filtering method to remove probes which displayed intensity levels similar to or below a threshold. Intensity levels detected for chromosome Y-specific probes in female samples are deemed to be background noise 20. To further minimize or remove nonbiological technical variations, such as batch effects caused by combining multiple datasets together, we applied ComBat using R package (sva_v3.4.0) to the pooled mRNA abundance levels 21; the sources of data were treated as batch effects and tumor grade was used as a covariate in ComBat.

**Table 1:** A list of datasets used in this analysis as well as their summary clinical information

Datasets	Number of patients	Number of genes	Median survival (years)	Median age	Median stage	Median grade	PMID	Sources
Berchuck	11	12080	NA	52	I	2	15897565	http://data.cgt.duke.edu/clinicalcancerresearch
Bild	112	12080	2.83	59	III	2	16273092	http://www.ncbi.nlm.nih.gov/geo/query/acc.cgi?acc=GSE3149
Denkert	68	12080	2.88	NA	III	3	19294737	http://www.ncbi.nlm.nih.gov/geo/query/acc.cgi?acc=GSE14764
Tothill	143	18989	2.42	59	III	3	18698038	http://www.ncbi.nlm.nih.gov/geo/query/acc.cgi?acc=GSE9899

### Differential expression analysis

To identify which genes were differentially expressed between different tumor grades, we analyzed the gene expression values across patient groups using a per-gene multivariate linear model. The expression levels were modeled as a function of tumor grades and the dataset of origin as:

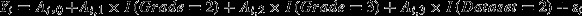
1where *Y*_*i*_ is the normalized mRNA abundance levels for the *i*th gene, A_i,0_ represents the baseline expression of the ith gene, *A*_*i*,1_ and A_i,2_ are the coefficients of tumor grade for the *i*th gene, Grade is an indicator where 0/1 represents the different grades, A_i,3_ is the coefficient of dataset for the *i*th gene, Dataset is an indicator where 1/2 indicates the different datasets, *ε*_*i*_ is an error term.

To test whether the difference in mRNA abundance levels between different tumor grade groups was statistically significant from zero, a model-based *t*-test was used (mRNA abundance levels from patients with grade 1 tumors were compared to those with grade 2 tumors and so forth). *P*-values were adjusted for multiple testing using false-discovery rate (FDR) correction 22. Coefficients representing the change in mRNA abundance levels between each comparison were adjusted with an empirical Bayes moderation of the standard error 23. Genes below a FDR threshold of 10% (i.e., *P*_adjusted_ < 0.1) were deemed significant; this threshold was chosen as the number of differentially expressed genes started to plateau across all group comparisons at thresholds lower than this value.

### Data visualization

Unsupervised machine learning was performed using divisive hierarchical clustering with the Divisive Analysis Clustering (DIANA) algorithm and Pearson's correlation as a similarity metric. We performed variance filtering on mRNA abundance levels with a threshold of 1. This filtering removed genes that were not differentially expressed. This analysis used the cluster (v1.14.4), lattice (v0.20-15), and latticeExtra (v0.6-24) packages from R statistical environment (v2.15.3). Venn diagrams were created using the VennDiagram package (v1.6.0) 24. An FDR-adjusted *P*-value (*P*_adjusted_) sensitivity plot was generated by plotting the number of genes altered at every *P*_adjusted_ value cut-off, with *P*-value thresholds spanning the range from *P*_adjusted_ = 1 × 10^−6^ to *P*_adjusted_ = 0.5.

### Pathway analysis

To identify pathways or biological functions associated with differentially expressed genes, we conducted pathway analysis using GoMiner 25 and Gene Ontology (GO) annotation 26. A relaxed *P*_adjusted_ cut-off of 0.25 was selected to obtain a list of genes that showed differential mRNA abundances between tumors of different grades. GoMiner analysis was run on the 2011-01 database built with the following settings: 10% FDR threshold, 1000 randomizations, all human databases and look-up options, the smallest category size for category statistics of 5 and all GO evidence codes, and ontologies.

### Survival analysis

To characterize the clinical utility of genes showing differential expression across tumors from different grades, we explored the prognostic ability of these genes to accurately predict patient survival. Patients were median-dichotomized based on mRNA abundance levels of those genes determined to be differentially expressed between tumor grades. Median dichotomization was performed separately for each dataset and a Cox proportional hazards model adjusted for tumor stage was then fit on the resulting data 27. Patient survival was modeled as a function of this group assignment. Survival analysis was conducted using the survival package (v2.37-4) in the R statistical environment.

## Results

### Global patterns of mRNA abundance

Four separate datasets of serous ovarian cancer were compiled, providing abundance measurements for 12,080 genes across 334 patients. Each dataset was normalized independently and then merged into a single dataset. Surrogate-Variable Analysis using the ComBat algorithm was performed to reduce batch effects (Fig. S1A and B show strong dataset-specific effects prior to batch-effect removal).

**Figure 1:**
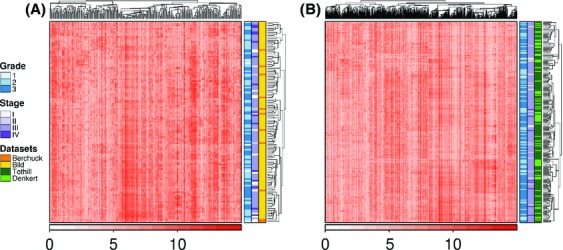
mRNA abundance levels of high-variance genes. Hierarchical clustering of mRNA abundance levels from ovarian cancer tumors shows that mRNA abundances are neither associated with clinical covariates (grade, stage) nor dataset of origin. (A) Datasets graded using the FIGO system (adjusted rand index [ARI] for grade, −0.006 [Grade]; dataset, −0.036; stage, 0.013). (B) Datasets graded using the Silverberg system (ARI for grade, −0.006; dataset, 0.024; stage, −0.015).

Hierarchical clustering of mRNA abundance levels using DIANA revealed minimal molecular differences based on clinical and technical covariates (grade, using the FIGO (Fig.1A) or Silverberg (Fig.1B) grading systems, stage, and dataset). This clustering effect was quantified using the adjusted rand index (ARI; no variable was deemed significant [all variables produced an ARI close to 0]). This confirms that mRNA abundances varied substantially even among tumors of the same histologic stage and grade 28.

### Genes associated with tumor grade

**Figure 2:**
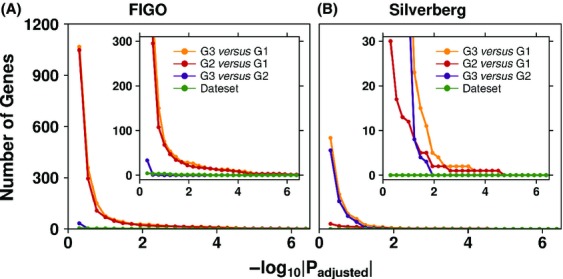
Adjusted *P*-value sensitivity analysis. The number of genes that showed differential abundance levels between each grade group comparison was calculated for different adjusted *P*-value cut-offs (*P*_adjusted_) for both the (A) International Federation of Gynecology and Obstetrics (FIGO) and (B) Silverberg grading systems.

We then sought to determine the number of genes differentially expressed between tumors of different grades. Since two different grading systems were used, we analyzed the FIGO and Silverberg grading systems separately using general linear modeling with multiple-testing correction. Surprisingly, very few genes were differentially expressed between FIGO grade 2 and 3 tumors, suggesting that these two groups are essentially indistinguishable. By contrast, FIGO grade 3 and 2 tumors both differed substantially from grade 1 tumors (Fig.2A). A slightly larger number of genes were differentially expressed between grade 3 and 1 tumors than between grade 2 and 1 tumors (75 vs. 69, respectively, at a 10% FDR cut-off); these findings were threshold independent. By contrast, Silverberg grade 1, 2 and 3 tumors differed from one another in all pair-wise combinations (Fig.2B). These results suggest that Silverberg grade 2 tumors (but not FIGO grade 2 tumors) comprise a molecularly distinct entity. Table S1 gives the gene-level results of our statistical modeling for all comparisons.

**Figure 3:**
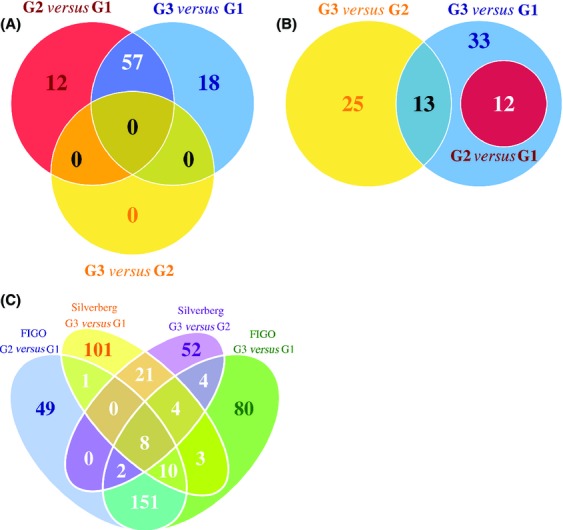
Overlap of genes with differential abundance between different grades of tumors. Differentially abundant genes between each pair of grades were compared for each of the (A) International Federation of Gynecology and Obstetrics (FIGO) and (B) Silverberg grading systems, and (C) across the entire dataset. (A) For FIGO graded tumors, no genes were differentially expressed between grade 2 and 3 tumors at a threshold of 10% false-discovery rate (FDR) while substantial overlap was observed between grade 3 versus 1 tumors and grade 2 versus 1 tumors. (B) A progressive pattern was observed for the Silverberg grading system. (C) Distinct groups of differentially abundant genes (at a FDR threshold of 25%) were observed, dependent on grading system used.

To determine whether this difference between grading systems held true at the level of individual genes, we chose a 10% FDR cut-off to identify differentially expressed genes. For FIGO graded tumors (Fig.3A), there were no differentially expressed genes between grades 2 and 3, even at this relaxed significance threshold. By contrast, both grade 2 and 3 tumors showed similar differences relative to grade 1 tumors (57 genes in common). Alternatively, tumors graded using the Silverberg system (Fig.3B) showed a more progressive pattern, where all genes differing between grades 1 and 2 also differed between grades 1 and 3. These data are consistent with the idea that FIGO grades 2 and 3 are molecularly indistinguishable, whereas Silverberg grading represents a spectrum of states, and that the two systems are characterized by distinct molecular features (Fig.3C). Furthermore, we found that grade was not associated with molecular subtype (Tothill dataset; *P* = 0.368; Pearson's Chi-square test).

### Pathway-level differences associated with tumor grade

**Figure 4:**
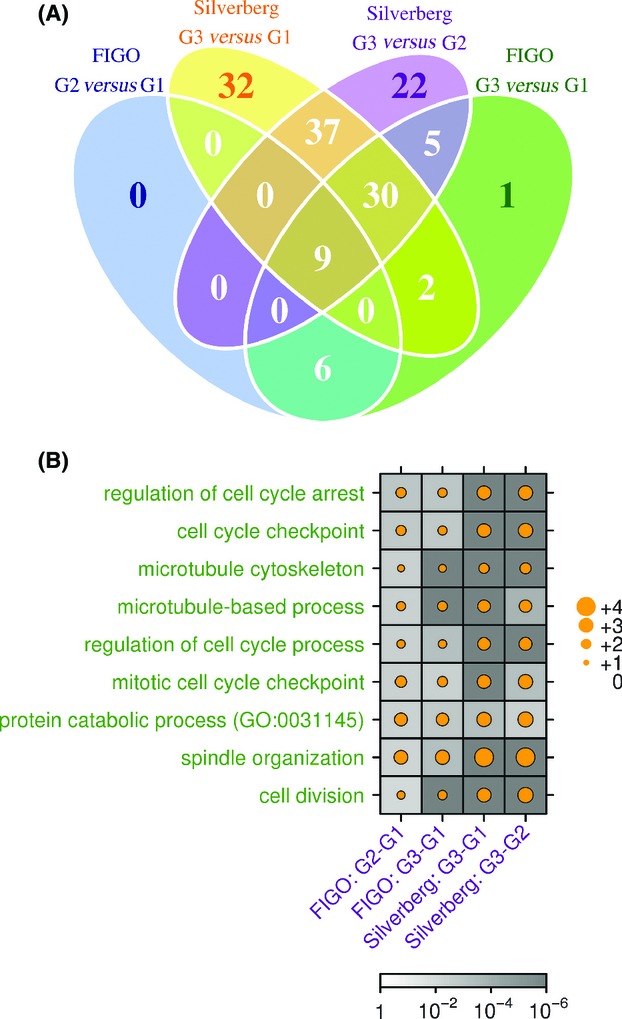
Overlap of enriched GO terms between different grading systems and different tumor grade comparisons. (A) Genes that showed differential expression at a relaxed *P*-value threshold of 25% false-discovery rate (FDR) were used for GoMiner analyses. GO terms were then filtered based on their FDR values (1% FDR threshold) and these terms were compared across different grade comparisons. (B) A total of nine GO terms were commonly enriched across all comparisons. Gray shaded boxes represent FDR values (darker shade for increased statistical significance); circle size represents log_2_ enrichment.

To identify the biological pathways altered by or governing the morphological differences between tumors of different grades, we performed GoMiner analysis on differentially abundant genes at a relaxed FDR cut-off of 25%. At a 1% FDR cut-off, 53 and 137 GO terms were significantly enriched across the FIGO and Silverberg datasets, respectively (Fig.4A). As nine GO terms were significantly enriched across all comparisons, they were further explored (Fig.4B). These terms include key cancer-related processes associated with rapid cell division, including cell cycle regulation and cytoskeletal and spindle organization.

### Differentially expressed genes predict survival

Previously, it was shown that grade did not provide additional prognostic ability independent of cell type or stage. To examine whether this was true of our data, we performed survival analysis for each dataset, using grade as the grouping variable (Fig. S2A, S2B, and S2C for Bild, Denkert, and Tothill datasets, respectively). We found that grade did not provide sufficient prognostic ability. As such, we chose to examine whether genes differentially expressed between tumors of different grades carry significant prognostic information. To test this effect, we modeled overall survival (OS) as a function of mRNA abundance levels. As described in the Materials and Methods section, patients were median-dichotomized to low and high expression based on the mRNA abundance levels of differentially expressed genes. A list of genes and their stage-adjusted hazard ratios and Cox proportional hazards model *P*-values and *q*-values (adjusted for multiple testing using FDR) are listed in Table S1. Although we did not observe enrichment for prognostic information in these differentially expressed genes (Table S2), we did identify six genes (*APOBEC3C*,*C11orf16*,*C21orf2*, *MUC5AC*, *SRD5A2*, and *TUBA4B*) that showed consistent prognostic abilities (Fig. S3).

## Discussion

Low-grade serous tumors are uncommon, accounting for less than 10% of ovarian serous carcinomas, and show morphologic progression from cystadenoma/adenofibroma to borderline serous tumor to micropapillary borderline tumor and finally to invasive low-grade serous carcinoma 29. This histological sequence is mirrored by progressive allelic imbalances: KRAS, BRAF, and ERBB2 mutations are identified in 2/3 of cases and p53 mutations are rare. In contrast, high-grade serous carcinomas frequently harbor p53 and BRCA mutations and lack the characteristic mutations of their low-grade counterparts. These tumors demonstrate a high level of chromosomal instability even in early stage cases and the majority likely arises from tubal intraepithelial carcinoma 30.

From a clinical perspective, patients with low-grade serous carcinoma are younger (median age at diagnosis 43 vs. 63 years) 31, but are more likely to manifest resistance to standard chemotherapy regimens 32. The binary low-/high-grade categories of the Malpica system are effectively nominal categories reflecting these distinct biological entities rather than grades of the same tumor 11. It has recently been questioned whether it is relevant to subclassify high-grade serous carcinoma into moderately and poorly differentiated categories.

In a study by Malpica, all Silverberg grade 1 and grade 3 tumors corresponded to low- and high grade, respectively 11 while 82% of Silverberg grade 2 tumors were high grade. The FIGO grading system was more heterogeneous: 97% of FIGO grade 1 was determined to be low grade, while the remaining 3% (1 case) was high grade. All FIGO grade 3 cases and 72% of FIGO grade 2 cases were high grade. Thus, the moderate category in these two systems seems to constitute a mix of high- and low-grade cases.

Stratification according to grade should reflect therapeutic, prognostic, or biological differences within a nominal diagnostic category. Previous study did not demonstrate prognostic differences between Silverberg grade 2 and grade 3 serous carcinomas 33. It has also been suggested that further stratification of high-grade serous carcinomas into FIGO moderately and poorly differentiated subsets is not clinically relevant based on similar TP53 mutation results and drug sensitivities 33. Vang and colleagues recommended additional molecular studies comparing morphologic subdivision within the high-grade category of serous carcinoma 10.

Our current work indicates that there is no significant difference in mRNA profiles of FIGO grade 2 and grade 3 ovarian serous carcinomas. In addition we have demonstrated distinct molecular characteristics between tumors graded with FIGO and Silverberg systems. Tumors graded with the FIGO system showed consistent results with what we would expect from a two-tier system, demonstrating greater molecular similarity between grade 2 and 3 tumors and more differences with grade 1 tumors. Results were different for Silverberg graded tumors, where similar numbers of molecular changes were observed between each pair of tumor grades. This discrepancy could in part be explained by the difference in the criteria used for grading. The FIGO grading system is based primarily on the percentage of solid cell architecture, whereas Silverberg system is based on the scores of three components: architecture, degree of nuclear atypia, and mitotic index 10. It remains possible that the more stringent scoring metric employed by the Silverberg system produced more biologically relevant results.

A similar analysis has previously been performed by Meingold-Heerlien and colleagues; 12,500 genes were profiled across tumors from 52 patients, including 44 with serous carcinomas (G1, *n* = 7; G2, *n* = 17; G3, *n* = 20), although they did not specify which system was used for grading 34. They identified a conspicuous distinction between low malignant potential (LMP)/G1 tumors and G2/G3 tumors. Statistical analysis found few differences between LMP and G1 tumors, but many more between G2 and G3, and large differences between LMP/G1 and G2/G3.

The Tothill dataset analyzed in this study was initially used to identify novel molecular subtypes of high-grade ovarian serous carcinoma 16. Tothill and colleagues identified unique molecular subtypes of high-grade serous carcinoma—C1 (high stromal response), C2 (high immune signature), C3 (high protein kinase expression), C4 (low stromal response), C5 (mesenchymal, low immune signature) subtypes, and C6 (low grade endometrioid). These molecular subtypes were randomly distributed between grade 2 and 3 tumors and univariate analysis showed significant differences in both progression-free survival (PFS) and OS. Multivariate analysis showed that the C1 group had a significantly worse outcome even when considering other known prognostic indicators such as stage, grade, age, and residual disease (PFS, *P* = 0.012; OS, *P* = 0.034) compared to the other subsets.

In the current study, no central pathologic review across the datasets was performed, hence some differences that we observed might potentially be due to misclassification of tumor grades; additional studies in consistent cohorts are needed to further validate the results. Furthermore, work on the identification of molecular signatures of ovarian cancer as well as characterization of single nucleotide variants (SNVs) and copy number aberrations (CNAs) will also be a valuable follow-up to the current study. As well, it will add great value if multiple grading systems are used within a single dataset and molecular differences assessed between the different systems. Nevertheless, we have shown in this study that FIGO-graded tumors exhibited great molecular similarities between grade 2 and 3 tumors, whereas Silverberg graded tumors demonstrate more diverse profiles between differentially graded tumors. Histologic grade carries clinical utility but more studies are needed to understand the biological processes in tumors; nevertheless, these data suggest that a two-tier grading system may be a preferred scheme for grading ovarian carcinoma of the serous subtype. This issue certainly merits additional exploration.

## Supporting Information

**Figure S1.** mRNA abundance levels prior to ComBat adjustment. mRNA abundance profiles were examined using DIANA clustering for each of the (A) FIGO and (B) Silverberg grading systems. Before ComBat, mRNA abundance levels in FIGO-graded tumors did not demonstrate strong dataset-specific bias (adjusted Rand index = −0.03), but expression levels in Silverberg graded tumors showed distinct patterns between the two datasets (ARI = 1).

**Figure S2.** Overall survival outcome. Difference in overall survival between low-grade (grade 1) and high-grade (grade 2 and 3) ovarian carcinoma patients was marginally significant for the (A) Bild dataset but was insignificant for both the (B) Denkert and (C) Tothill datasets, suggesting that histologic grade as a clinical covariate has minimal prognostic ability.

**Figure S3.** Gene-specific survival outcome over time. Genes that showed consistent differential expression between grade 2 and 1 tumors and grade 3 and 1 tumors were assessed for their prognostic ability. Patients were median-dichotomized into low- and high-expression groups based on the mRNA abundance levels of these genes. Hazard ratio (HR) indicates the ratio of hazard rates between patients with high expression level of a given gene and those with a low expression level. The numbers in the bracket following HR denote the 95% confidence interval for the hazard ratio, which are derived from the standard deviation of the regression model. Genes with *P* ≤ 0.05 (Wald test) demonstrate strong prognostic ability.

**Figure S4.** Power calculation in current cohort. Power calculation for effect sizes ranging from 0 to 3.2 for both FIGO (left) and Silverberg (right) graded tumors. Dashed horizontal line represents a threshold of 80% power.

**Table S1.** A list of differentially expressed genes between different tumor grades and their prognostic abilities.

**Table S2.** Proportion of significantly prognostic genes across different comparison groups.

**Table S3.** Sample summary broken down by grading system and different tumor grades and the statistical power for each comparison. In datasets graded using the FIGO system, we have 80% power to detect an effect size of 2.34 for grade 3 versus grade 1 comparison, 2.35 for grade 2 versus grade 1 comparison, and 0.90 for grade 3 versus grade 2 comparison. Similarly, in datasets graded using the Silverberg system, we have 80% power to detect an effect size of 2.22 for grade 3 versus grade 1 comparison, 2.31 for grade 2 versus grade 1 comparison, and 0.71 for grade 3 versus grade 2 comparison.
