# Supplementary figures and images for: The transcriptomic profile of ovarian cancer grading
Source: Cancer Med. 2014 Oct 15;4(1):56–64. doi: 10.1002/cam4.343 (PMC4312118; doi:10.1002/cam4.343)

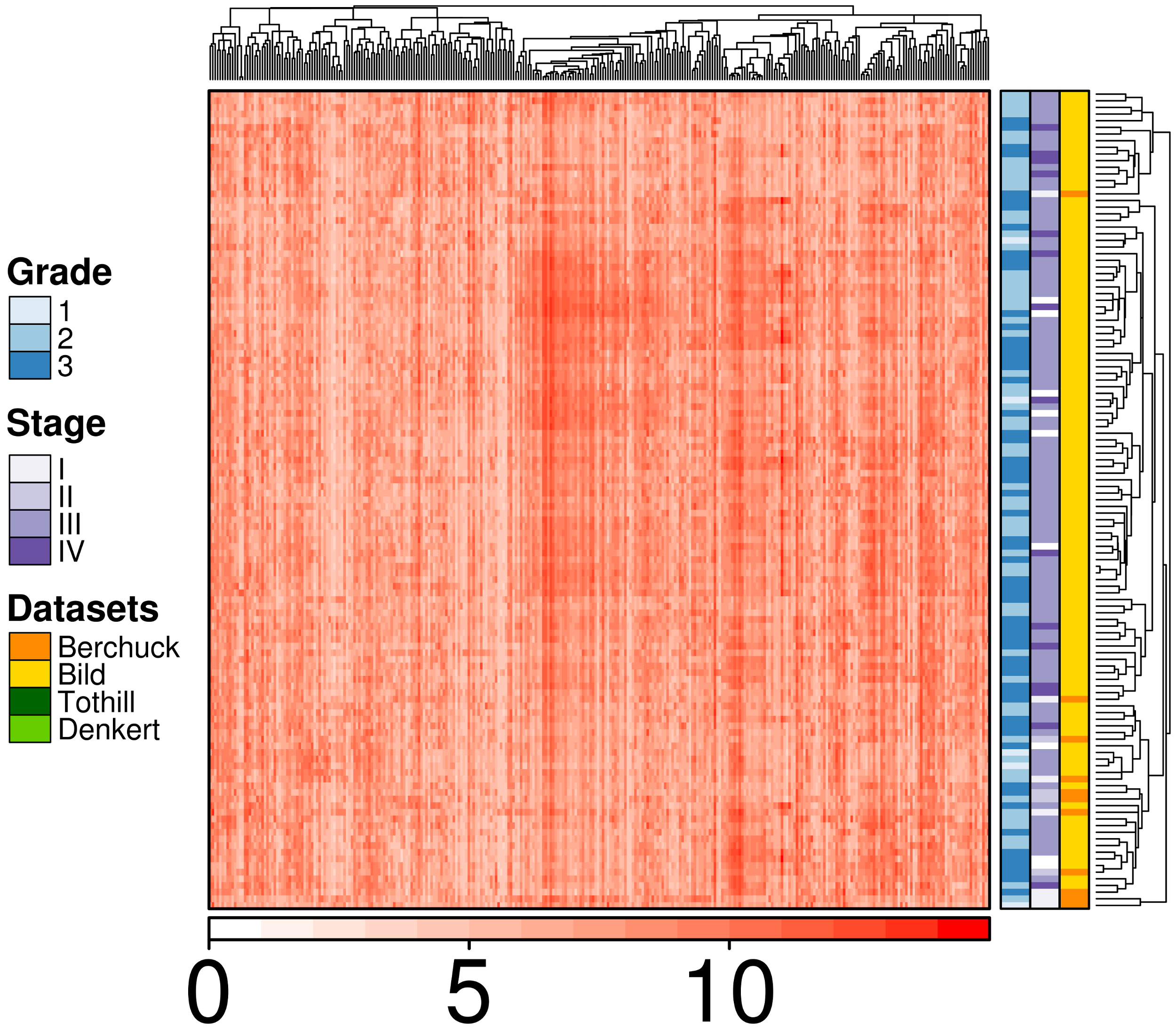

Supplement: Supplementary file 1 [file cam40004-0056-sd1.tif]

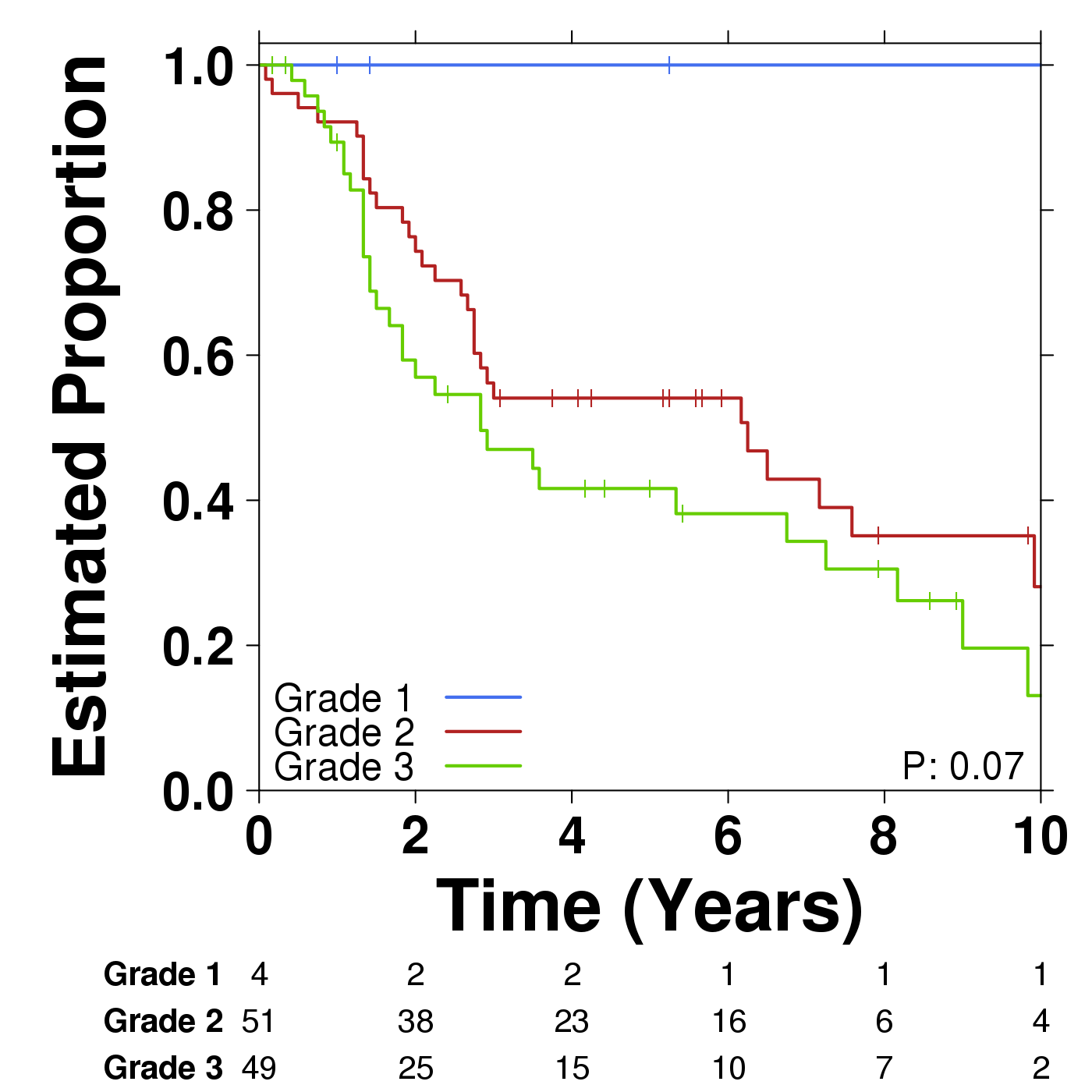

Supplement: Supplementary file 2 [file cam40004-0056-sd2.tif]

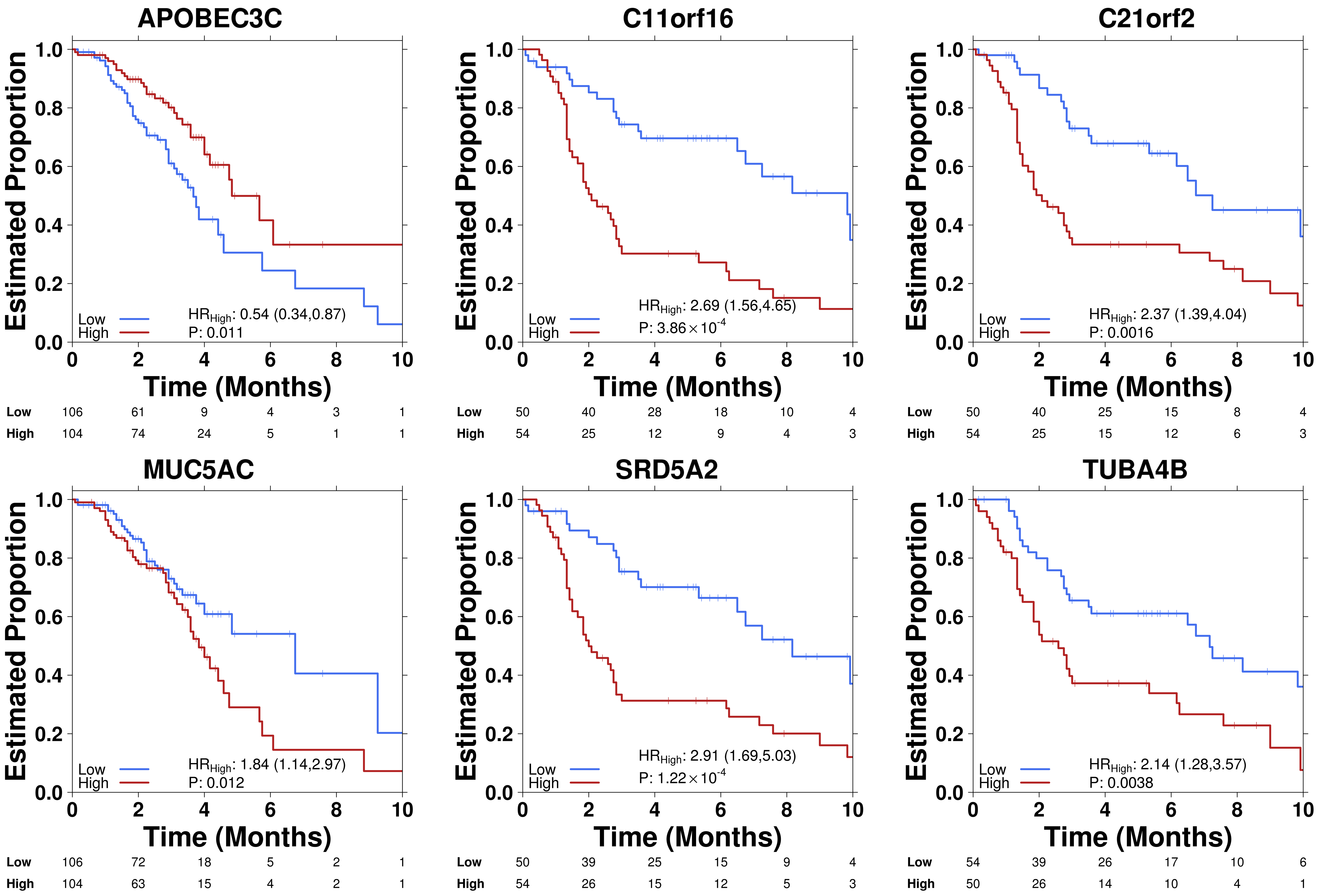

Supplement: Supplementary file 3 [file cam40004-0056-sd3.tif]

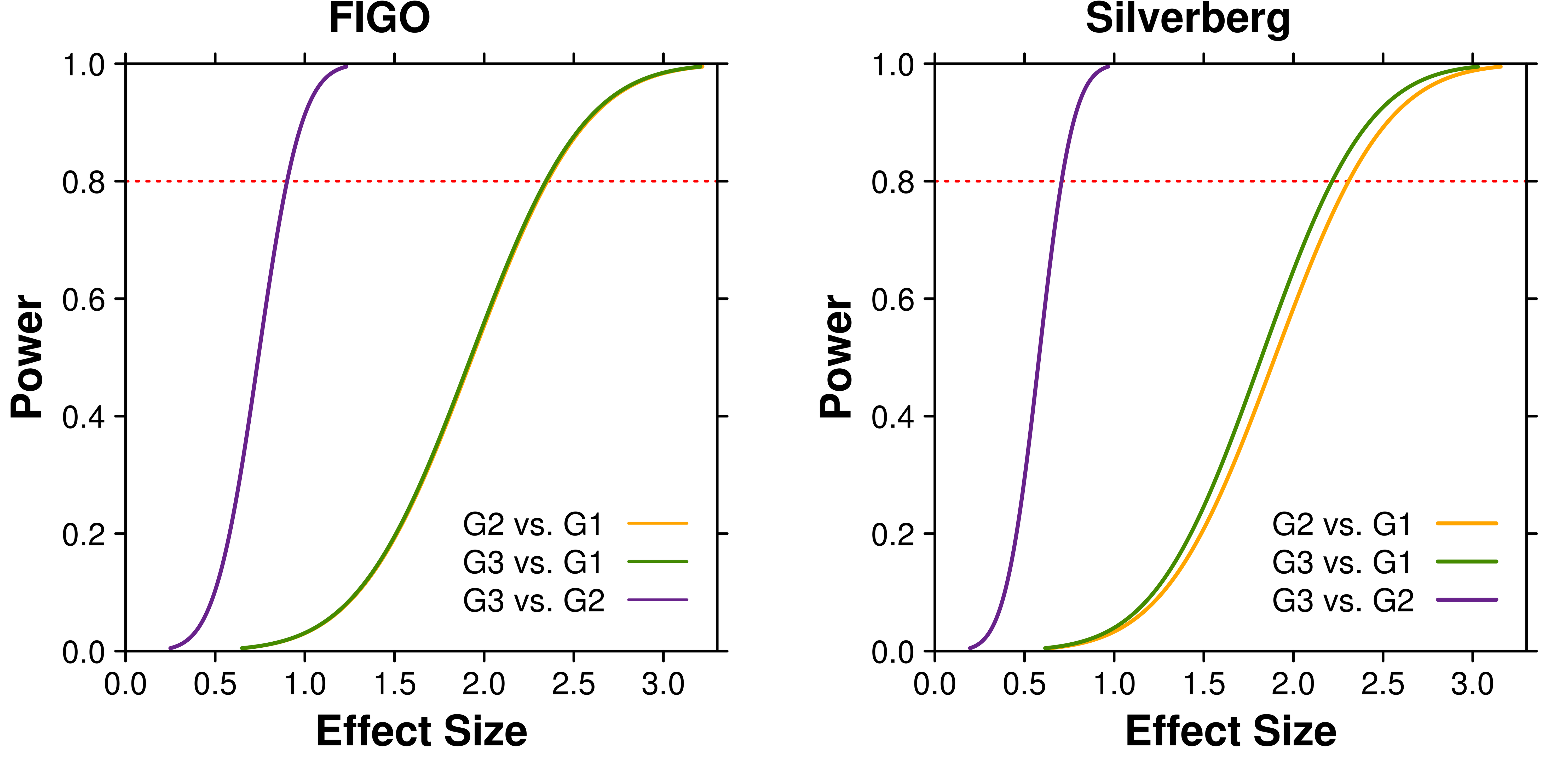

Supplement: Supplementary file 4 [file cam40004-0056-sd4.tif]
